# Bioactive Peptides in Animal Food Products

**DOI:** 10.3390/foods6050035

**Published:** 2017-05-09

**Authors:** Marzia Albenzio, Antonella Santillo, Mariangela Caroprese, Antonella della Malva, Rosaria Marino

**Affiliations:** Department of Agricultural Food and Environmental Sciences (SAFE), University of Foggia, Via Napoli 25, 71122 Foggia, Italy; antonella.santillo@unifg.it (A.S.); mariangela.caroprese@unifg.it (M.C.); antonella.dellamalva@unifg.it (A.d.M.); rosaria.marino@unifg.it (R.M.)

**Keywords:** milk, cheese, meat, bioactive peptides, human health

## Abstract

Proteins of animal origin represent physiologically active components in the human diet; they exert a direct action or constitute a substrate for enzymatic hydrolysis upon food processing and consumption. Bioactive peptides may descend from the hydrolysis by digestive enzymes, enzymes endogenous to raw food materials, and enzymes from microorganisms added during food processing. Milk proteins have different polymorphisms for each dairy species that influence the amount and the biochemical characteristics (e.g., amino acid chain, phosphorylation, and glycosylation) of the protein. Milk from other species alternative to cow has been exploited for their role in children with cow milk allergy and in some infant pathologies, such as epilepsy, by monitoring the immune status. Different mechanisms concur for bioactive peptides generation from meat and meat products, and their functionality and application as functional ingredients have proven effects on consumer health. Animal food proteins are currently the main source of a range of biologically-active peptides which have gained special interest because they may also influence numerous physiological responses in the organism. The addition of probiotics to animal food products represent a strategy for the increase of molecules with health and functional properties.

## 1. Introduction

The general consensus on the impact of lifestyle on human health considers that diet represents a crucial factor in terms of human health status. Proteins of animal origin have been recognized for their nutritional properties as an essential source of amino acids upon digestion, but both digestion and industrial processing may liberate peptides from the parent protein which have biological functions. Animal food products, in particularly dairy foods, were characterized by genetic polymorphisms of the main proteins that impact on protein hydrolysis during food processing prior to consumption and digestion in the human organism. Biologically-active peptides can be produced from milk proteins through different pathways involving milk secretion, milk storage, milk processing, and milk digestion due to enzymatic hydrolyses by indigenous enzymes, digestive enzymes, and microbial enzymes from starter and non-starter cultures. The integrity and structure of meat proteins undergo changes during rigor mortis, the resolution of rigor mortis, and long-term frozen storage. Particularly, a large number of peptides showing important physiological activities are released during meat processing. Dietary supplements allow the delivery of positive molecules in dosages that exceed those obtained from conventional food products. However, great interest has been observed regarding the bioactive components naturally contained in foods which have an impact on biological processes. Bioactive components in foods represent dietary elements that impart a measurable biological effect that affect health in a beneficial way, such as immune-modulating, antihypertnesive, osteoprotective, antilipemic, opiate, antioxidative, and antimicrobial activities [1]. The in vitro bioactivities of food components have been widely explored and the present effort is to study their effects in vivo on healthy subjects or patients with different pathologies. This review provides an integrated overview on the occurrence of bioactive peptides in animal food products, and the role on human health of milk and meat peptides is also discussed.

## 2. Role of Dairy Proteins on Human Health

Protein is a very heterogeneous component in cow, sheep, and goat milk, mainly influenced by genetic variants. The genetic polymorphisms of milk proteins are of importance as they are associated with quantitative and qualitative parameters in milk. In particular, genetic polymorphism was associated with different levels of protein synthesis in milk, different rates of phosphorylation and glycosilation of the peptide chain, and amino acid sequences of the protein [2]. In cattle, the six main milk proteins are encoded by highly-polymorphic genes with up to 47 protein variants identified, affecting not only the specific protein expression and, as a consequence, milk composition and cheesemaking, but they are also involved in various aspects of human nutrition [3,4]. A recent review paper [5] deepened the presence of a complex polymorphism at casein loci levels in small ruminant species and its role on the nutritional properties on milk and dairy products. In this contest the polymorphisms of milk proteins from small ruminant species have significant potential in human pathology. Genetic polymorphisms of milk proteins also play an important role in eliciting different degrees of allergic reaction [6,7,8]. Caseins, and especially α-CN, are among the most important milk allergens [9,10,11]. Milk from other species alternative to cow has been investigated for its role in children with cow milk allergy (CMA); higher TNF-α levels were indeed found after exposure to cow milk casein and β-Lg than after exposure to the same fractions from goat milk [12]. Some studies have indicated an unusually high incidence of allergenic illnesses in those suffering with epilepsy [13]. Most of the authors examined the relationship between food allergy and epilepsy by comparing groups of adult patients with healthy control subjects [14]. Cytokine productions by cultured PBMCs from infants with generalized epilepsy was influenced by protein fractions of milk from bovine, caprine, and ovine species. PBMC’s ability to secrete cytokines in response to milk and protein fraction stimulation may be a predictor of the secretion of pro- and anti-inflammatory cytokines in the bloodstream of the challenged patients [14].

### 2.1. Occurrence of Bioactive Peptides in Milk

Biologically-active peptides can be produced from milk proteins through different pathways involving the action of indigenous enzymes, digestive enzymes, and microbial enzymes from starter and non-starter cultures acting during milk secretion, milk storage, milk processing, and milk digestion. Proteolytic activity in fresh raw milk is attributed to indigenous and microbial enzymes. Among the indigenous enzymes, milk contains at least two main proteinase systems, the plasmin-plasminogen system and lysosomal enzymes, as well as possibly other proteolytic enzymes. Plasmin is the principal proteolytic enzyme in raw milk and is associated with casein micelles. The second proteinase in milk is cathepsin D, activity of which is significantly correlated with somatic cell count, which contains several proteinases, including cathepsin B, L, and G, and elastase [15]. The principal indigenous proteolytic enzymes were investigated and characterized in ovine and caprine milk [16,17,18,19,20]. Some bioactive peptides found in milk and dairy products and their functionality have been reported in Table 1. Indigenous enzymes play a role in the liberation of bioactive peptides during milk secretion and storage. A great number of peptides were found in goat milk incubated up to seven days without any protease inhibitors; plasmin was shown to play a major role in the hydrolysis of casein and high numbers of peptides were derived from the hydrolysis of β-casein. Almost 90% of the peptides identified shared a structural homology with previously-described bioactive peptides in caprine and bovine milk and dairy products showing encrypted sequences of bioactive peptides able to exert ACE-inhibitory activity [21,22]; antihypertensive activity [22,23], and antioxidant activity [24].

In sheep milk, several peptides with functional activity were found deriving from the action of peptidases of different origins on casein fractions. At least three ACE inhibitory peptides were liberated by purified proteinase of *Lb helveticus* [26] from αs1- and αs2-caseins, and antihypertensive and antioxidant peptides were found in ovine sodium caseinate incubated with *Bacillus* sp. P7 [35]. Four antibacterial peptides were identified from a pepsin hydrolysate of ovine αs2-casein [27], corresponding to αs2-casein fragments f(165–170), f(165–181), f(184–208), and f(203–208), with the former being most effective against Gram-negative bacteria. The peptide corresponding to ovine αs2-casein f(203–208) is a good example for a multifunctional peptide, because it exhibited not only antimicrobial activity, but also potent antihypertensive and antioxidant activity [36].

The most common way to produce bioactive peptides is through enzymatic hydrolyses of whole protein molecules: digestive enzymes and different enzyme combinations of proteinases, including alcalase, chymotripsin, pancreatin, pepsin, and thermolysin have been utilized to generate bioactive peptides from various proteins [37]. Ingested proteins undergo different stages of gastrointestinal hydrolysis in the stomach and intestinal lumen due to proteinases, such as pepsin, trypsin, and chymotripsin. Finally, these peptides are further digested by brush border peptidases at the surface of intestinal epithelial cells to produce amino acids and oligopeptides able to undergo the absorption process. For example, β-casomorphins and phosphopeptides derived from casein (CPPs) are produced in vivo during digestion of dairy products, including milk, fermented milk, cheese, and yogurt [38]. The quantity of peptides released upon digestion is hardly predictable and, consequently, the beneficial effects of human health. Peptide bioavailability is dependent on the resistance of the peptide to hydrolysis in the gastrointestinal tract and serum and its ability to be absorbed across the intestinal epithelium [39]. However, some authors report that the potential yield of bioactive peptides, during the digestion of the major dairy proteins, is relatively high. Meisel and Fitzgerald [40] estimated the theoretical yield of opioid peptides encrypted in milk proteins ranged between 2% and 6%.

### 2.2. Occurrence of Bioactive Peptides in Dairy Products

The ripening process in cheese encompasses several biochemical pathways dealing with the proteolytic, lipolytic, and glicolytic processes. Many dairy cultures are highly proteolytic, leading to bioactive peptide accumulation in ripened dairy products. Depending on the type of dairy products the level of peptides naturally formed in the matrix varies along with the equilibrium between the liberation and the further hydrolysis during ripening. However, the bioactive peptides have been characterized in a wide variety of dairy products distinguished on the basis of the time of ripening in fresh, short, and long ripened cheese, and on the basis of the technological process of fermented cheese, pasta filata cheese, and cooked cheese.

In long-ripened Gruyere de Comté and Cheddar cheese CPPs naturally occurred due to the primary action of chymosin and plasmin and further hydrolysis of endopeptidases from non-starter lactic acid bacteria [29,41]. The maximum ACE-inhibitor activities were found in Gouda cheese ripened for three months than in short- and long-ripened cheese. On the contrary, in Manchego cheese, from ovine milk, the ACE-inhibitory activity showed a different and complex evolution along with the ripening time decreasing in the first four months, with a subsequent increase and then decreasing again in twelve-month cheese [30]. In Emmental cheese, different bioactivities were detected as mineral-carrying, antimicrobial, antihypertensive, and immunestimulatory due to both the action of plasmin and cathepsin D and to proteinases associated with microbial starter [32]. In Cheddar cheese, the sequence RPKHPIK was found in Festivo and Iberian ovine cheeses [42,43,44] and was also found when the cheeses were subjected to a hydrolysis process that simulated gastric digestion and reported antimicrobial activity. The sequence RPKHPIKHQ was found in water-soluble peptide preparation isolated from Gouda ripened for eight months, showing a potent antihypertensive activity tested in spontaneously-hypertensive rats [33]. Furthermore, the fragment 1–23 of αs_1_-CN, known as Isracidin, originated from the proteolytic activity of chymosin and exerted antimicrobial activity on several microorganisms [45]. The sequence PQEVLNENLLRF was referenced by Minkiewicz et al. [28] as an immunomodulating and antimicrobial peptide sequence in the primary structure of αs_1_-CN freed by chymosin activity. Furthermore, antimicrobial peptides were isolated from Mozzarella, Italico, Crescenza, and Gorgonzola cheeses [34] with a specific inhibitory action towards endopeptidase from *Pseudomonas fluorescens.* Such a microorganism is responsible for the impairment of technological and organoleptic features of dairy products. The fermented milks are a source of bioactive peptides with anticariogenic, antihypertensive, mineral binding, and stress relieving activities due to the action of probiotic strains such as *Lb. casei, Lb. helveticus*, and *S. cerevisiae* [46,47,48].

The development of probiotic cheeses regarded Cheddar cheese [42,49,50,51,52,53,54], Gouda cheese [55], Cottage cheese [56], Pategrás cheese [57], Crescenza cheese [58], Minas fresh cheese [59,60], and Turkish white cheese [61]. Few studies have been conducted on the production of functional cheeses made from ewe milk; the first research was performed on PDO Canestrato pugliese cheese using *B. bifidum* and *B. longum* [62] as a starter adjunct. Probiotics added to cheese yield a wide spectrum of enzymes able to influence the biochemical events involving the protein and lipid fractions in cheese during ripening. These events have an impact on the development of texture, flavor and health components of cheese. The use of lamb rennet paste containing probiotics is a suitable strategy for innovation in traditional ovine cheese without modification of the production procedures [19,63,64,65,66,67]. This could provide a spin-off for health properties of cheese and for its ripening features, such as an acceleration of the ripening process with economic advantages to producers. It was found that using starter cultures and *L. acidophilus* and *Bifidobacteria* spp. Produced ACE-inhibitory activity peptides in Festivo cheese [43] and Manchego cheese [31]; peptides with antimicrobial activity were found in Cottage cheese produced with *Bifidobacteria* [68]. In functional Scamorza cheese made from ovine milk, containing a mix of *B. longum*-, *B. lactis*-, and *L. acidophilus*-specific peptides deriving from microbial enzymes were found in cheese at fifteen days of ripening. Several fragments were identified which shared structural homology with previously-reported peptides with ACE-inhibitory activity, antimicrobial activity, antihypertensive activity, and immunomodulating activity. Specific peptides deriving from microbial enzymes may be regarded as tracing fragments and may represent a tool to verify the presence and activity of probiotic cultures in cheese. In functional Scamorza cheese fragments were identified deriving from β-galactosidase and from endonuclease associated to *B. longum*, or deriving from enzymes yielded by *Lactobacillus acidophilus*.

## 3. Bioactive Peptides in Meat and Meat Products

### 3.1. Bioactive Peptide Generation

Due to the presence of high-quality proteins, meat represents the most investigated source for the isolation of novel bioactive peptides. Different mechanisms concur for bioactive peptide generation from meat and meat products (Table 2). During meat post-mortem aging, the proteolytic activity due to endogenous enzymes (calpains and cathepsins) is a key process that affects the destructuration of proteins and, consequently, the production and release of a large number of peptides and free amino acids [69,70]. Bauchart et al. [71], in a study on aged beef, found an increase of bioactive peptides in meat after 14 days of post mortem storage than in fresh meat. In a recent study, Fu et al. [72], also demonstrated that post-mortem aging can generate bioactive peptides of about 3 kDa in longissimus dorsi and in semitendinosus muscles after 20 days of extensive proteolysis. During post-mortem meat storage the generation of peptides may also be driven by oxidation processes [73]. An oxidative status could regulate the endogenous enzymatic activity and, consequently, the myofibrillar and sarcoplasmic protein degradation [74]. Changes of temperature and pH can affect the content of bioactive peptides during meat storage due to the variation in the activity of endogenous enzymes and the destruction of pH or heat-sensitive amino acids [75,76].

It is known that bioactive peptides are generated naturally in mammals within the gastrointestinal tract during the metabolisms of dietary meat proteins [77,78]. During gastrointestinal proteolysis, ingested meat-derivative proteins are attacked by stomach-secreted digestive enzymes, such as pepsin, followed by trypsin, chymotrypsin, elastase, and carboxypeptidase secreted in the small intestine with a consequent generation of biological peptides [79]. For this reason, in order to generate potentially-functional peptides from meat products, the gastrointestinal digestive system has been simulated to generate peptides similar to those released in a physiological digestion process. The process that simulates the gastrointestinal digestion is based on an enzymatic hydrolysis using different commercial exogenous proteinases obtained from animal tissues (pepsin and tripsin), plants (papain, ficin, and bromelain), and microbial sources (alcalase^®^, flavourzyme^®^, neutrase^®^, collagenase, or proteinase K) [79,80,81]. Enzymatic hydrolysis is a widespread method selected by food and pharmaceutical industries to produce bioactive peptides. In addition to meat sources, several bioactive peptides have been obtained through enzymatic hydrolysis from meat collagen or slaughtered by-products (trimmings, organs, hemoglobin), as reported in many studies [73,82].

Other mechanisms, such as freezing and cooking processes, can affect the isolation and availability of bioactive peptides from meat. Freezing can denature proteins due to different chemical and physical stress mechanisms, including ice formation, pH variations, and cold temperature [99], leading to an increase of bioactive peptides. Cooking can affect the generation of peptides and their related bioactivities [72,76] due to changes in the native conformation (denaturation) and rupture of intramolecular forces of proteins caused by heat [100].

A number of bioactive peptides were shown to be released, also, from meat products during curing or ripening processes [101]. The proteolytic degradation that occurs during the ripening of dry-cured ham or during fermentation of sausages, responsible also for flavor and texture, lead to a production of small peptides and free amino acids [83,102]. In particular, in fermented meat products the protein degradation is influenced by different variables as product formulation, processing conditions, and the presence of starter cultures. The content of peptides is influenced by proteolytic degradation of endogenous enzymes together with lactic acid bacteria. In particular, the presence of lactic acid bacteria induces a decrease of pH resulting in a greater activity of endogenous muscle proteases [103].

### 3.2. Functionality of Meat Bioactive Peptides

Meat peptides have proven effects on consumer health due to different types of bioactivity, including antihypertensive, antioxidant, antithrombotic, antimicrobial, or anticancer activities [104]. Bioactivities of peptides depend on the sequence, amino acid composition, and molecular mass [105]. Furthermore, Vermeirssen et al. [39] reported that the length of peptides could affect the intensity of the bioactivity, with smaller peptides characterized by greater bioactivity.

The most extensively-studied meat bioactive peptides are the angiotensin I-converting enzyme inhibitory (ACE-I) peptides, probably due to their implication in the regulation of blood pressure. ACE is a dipeptidylcarboxypeptidase enzyme that convert angiotensin I (decapeptide) into angiotensin II (octapeptide) resulting in a vasoconstriction of the arteries and, consequently, an increase of blood pressure. Therefore, the inhibition of ACE could be linked to the prevention of cardiovascular disease [106]. Meat proteins are a good source of ACE-I peptides with in vitro and in vivo bioactivities. In recent years, several bioactive peptides have been isolated through the hydrolysis of meat proteins with gastrointestinal enzymes, like pepsin, trypsin, chymotrypsin, or pancreatin. Katayama et al. [84] found two different ACE-I peptides from pork meat (KRQKYD, EKERERQ) through pepsin treatment. Both isolated peptides were studied in vivo in rats showing a hypotensive activity after three and six hours of oral administration.

Twenty-two ACE-I peptides from pork meat using pepsin and pancreatin proteases were isolated in vitro. Among these, KAPVA and PTPVP peptide sequences showed the highest antihypertensive activity [85]. Subsequently, in 2012, the same authors [86] investigated, in vivo, the bioactivity of KAPVA, PTPVP, and RPR peptides in rats, highlighting a major decrease of blood pressure by KAPVA and PTPVP peptides than RPR sequence in rats after eight hours of oral administration.

Peptides extracted from connective tissue were also identified as inhibitors of ACE [92,93,107]. Gómez-Guillén et al. [108] reported that the bioactivities of collagen-derived peptides depends on the amount of Gly and Pro amino acids. In vitro and in vivo ACE-I properties were found in peptides isolated from hydrolysate of bovine Achilles tendon collagen with bacterial collagenase [92]. After hydrolysis, samples were purified, sequenced, and identified as AKGANGAPGIAGAPGFPGARGPSGPQGPSGPP and PAGNPGADGQPGAKGANGAP. Both peptides showed ACE-I activity after an oral administration in rats. In recent years, Fu et al. [72,107] also found bioactive peptides from collagen extracted derived both from nuchal ligament of bovine carcasses (GPRGF) and from cooked semitendinosus muscle (SPLPPPE, EGPQGPPGPVG, and PGLIGARGPPGP) showing greater ACE and renin-inhibitory activities. In addition, Saiga et al. [94] isolated peptides with in vivo ACE-I activity from chicken collagen after hydrolysis with a protease from *Aspergillus oryzae*.

Several peptides isolated from meat are characterized by an antioxidant activity due to their capability to inhibit lipid peroxidation, chelate metal ions, and remove free radicals and ROS [109,110]. The most important antioxidants naturally present in meat are carnosine and anserine dipeptides, which explicate their antioxidant activity chelating pro-oxidative metals [87]. In addition to the peptides that are naturally present in meat, peptides with antioxidant activity were also generated through the hydrolysis with specific proteases. Saiga et al. [87], in an in vitro study on porcine myofibrillar proteins hydrolyzed with papain and actinase E, found five peptides (DSGVT, IEAEGE, EELDNALN, VPSIDDQEELM, and DAQEKLE) that exhibited an antioxidant activity using the linolenic acid peroxidation system. The same authors suggested that the highest antioxidant activity was reached by the DAQEKLE peptide obtained by actinase E, corresponding to a part of the tropomyosin alpha-1 chain. Thus, the type and specificity of proteases used play an important role in determining the antioxidative properties of peptides. Furthermore, three peptides (ALTA, SLTA, and VT) obtained from porcine skeletal muscle actomyosin showed antioxidative activity not only in vitro, but also in vivo in rats [88]. Four antioxidant peptides were also obtained from porcine collagen by Li et al. [95] using three different protease treatments (pepsin and papain, protease from bovine pancreas, and a cocktail of protease from bovine pancreas, bacterial proteases from *Streptomyces*, and *Bacillus polymyxa*). Results of this study showed that collagen treated with the cocktail of three enzymes demonstrate higher antioxidant activity and a major number of peptides (QGAR, LQGM, LQGMH, and LC) rather than the other treatments. In recent years, Banerjee and Shanthi [92] isolated a 36-amino acid residue peptide with free radical scavenging and metal chelating properties from bovine tendon collagen α1. Peptides with antioxidant activity can be produced during meat processing. Twenty-seven antioxidant peptides were sequenced using LC-MS/MS in samples of Spanish dry-cured ham [96]; in this study the highest scavenging activity was identified in the two different peptides (SAGNPN and GLAGA). Broncano et al. [98] also isolated two peptides (FGG and DM) with antioxidant activity in pork Chorizo sausages. Recently, Xing et al. [97] purified several antioxidant peptides from dry-cured Xuanwei ham, highlighting the highest antioxidant activity in DLEE peptide.

Peptides with antithrombotic properties were also isolated from meat. Morimatsu et al. [89] and Shimizu et al. [90] isolated peptides that exhibited antithrombotic activity from porcine longissimus dorsi muscle hydrolyzed with papain. Particularly, Shimizu et al. [90] tested the antithrombotic activity both in vitro, by a platelet function test using rat blood, and in vivo, by oral administration to mice (dose 70 mg/kg of body weight). In vivo results showed that the meat-derived peptide significantly reduced carotid artery thrombosis and decrease platelet activity with a comparable effect to aspirin treatment (at a dose of 50 mg/kg of body weight).

Although a number of peptides with antimicrobial activity have been isolated from bovine blood, only one study showed the presence of antimicrobial peptides derived from bovine meat [91]. In this study, Jang et al. [91] isolated four peptides (GLSDGEWQ, GFHI, DFHING, and FHG) after the hydrolysis with commercial enzymes of beef sarcoplasmic proteins. All peptides were subsequently tested for antimicrobial activity against six pathogens (*Escherichia coli*, *Pseudomonas aeruginosa*, *Salmonella typhimurium*, *Staphylococcus aureus*, *Bacillus cereus*, and *Listeria monocytogenes*). Results showed a different antimicrobial effect against one, or more, bacteria. In particular, GLSDGEWQ peptide showed an inhibition effect on *Escherichia coli*, *Salmonella typhimurium*, *Bacillus cereus*, and *Listeria monocytogenes*, while all tested peptides were found to be active against *Pseudomonas aeruginosa*.

It is known that some peptides can also exhibit anti-cancer activity, inhibit cell proliferation and have cytotoxic effects against cancer cells [111]. Jang et al. [91], investigated four peptides extracted from bovine sarcoplasmic proteins against breast, gastric, and lung adenocarcinoma. Results showed that the GFHI peptide had a greater cytotoxic effect against cancer cells of the breast and decreased the viability of gastric cells. In addition, an inhibitory effect on the proliferation of gastric cells has been found for the GLSDGEWQ peptide.

It is known that, after oral intake, bioactive peptides need to be absorbed intact to ensure their bioactivity within the cellular environments. In this regard, it is important that peptides enter the circularly system intact and remain active during the digestive process [112]. Small-sized peptides are more resistant to degradation by the intestinal enzymes and more easily absorbed to the circularly system [113]. Ohara et al. [114] detected small peptides derived from collagen in blood after oral ingestion of protein hydrolysate products. In recent years, nutrient absorption at the intestinal level is studied using an experimental model involving cultures of colon Caco-2 cells. Shimizu et al. [115] reported that chicken collagen octapeptide (GAXGLXGP) can be transported across a human intestinal epithelium. Recently, Fu et al. [107] also identified two peptides derived from bovine collagen (VGPV and GPRGF) with ACE-inhibitory activity into Caco-2 cells in the human intestinal epithelium, highlighting the bioavailability of these peptides.

Meat-derived bioactive peptides, due to their biological properties, are promising candidates as ingredients of functional or health-promoting foods [116]. Although the meat functional peptide-based products have not yet been commercialized by the industry, meat functional products could open a new market. In particular, development of functional fermented meat products could be a strategy to introduce to the market products with high nutritional value.

## 4. Occurrence of Bioactive Peptides in Egg

The avian egg is an important source of nutrients, containing all of the proteins, lipids, vitamins, minerals, and growth factors required by the developing embryo, as well as a number of defense factors to protect against bacterial and viral infection [117]. Especially, egg white contains a number of proteins with antimicrobial activities, including bacterial cell lysis, metal binding, and vitamin binding.

Lysozyme is well known to exert antimicrobial activity and, more recently, enzymatic hydrolysis of lysozyme has been found to enhance its activity by exposing antibacterial portions of the protein and producing peptides with antibacterial activity. Peptides corresponding to amino acid residues 98–112 [118], 98–108, and 15–21 [119] possessed antimicrobial activity against *E. coli* and *S. aureus*. Furthermore, peptides produced by the enzymatic digestion of ovalbumin, and their synthetic counterparts, were found to be strongly active against *Bacillus subtilus* and, to a lesser extent, against *E. coli*, *Bordetella bronchiseptica*, *Pseudomonas aeruginosa*, and *Serratia marcescens*, as well as *Candida albicans* [120].

Several egg white proteins and peptides have demonstrated immunomodulating activity. Tezuka and Yoshikawa [121] found that the phagocytic activity of macrophages was increased by the addition of ovalbumin peptides, OA 77-84 and OA 126-134, derived from peptic and chymotryptic digestions, respectively.

It has been reported that certain egg white-derived peptides can play a role in controlling the development of hypertension by exerting vasorelaxing effects [122]; a vasorelaxing peptide, ovokinin (OA 358-365), was isolated by the peptic digestion of ovalbumin. Additionally, a peptide produced by chymotrypsin digestion and corresponding to OA 359-364, was found to possess vasorelaxing activity. Both peptides were administered orally in spontaneously hypertensive rats and were found to significantly lower the systolic blood pressure. The replacement of amino acids in the ovokinin (2–7) peptide has resulted in enhanced antihypertensive activity, with the most potent derivative resulting in a 100-fold more potent antihypertensive activity [123]. Two angiotensin I converting enzyme (ACE)-inhibitory peptides were also identified in ovalbumin by peptic (OA 183-184) and tryptic (OA 200-218) digestions. Miguel et al. [124] examined peptides with ACE-inhibitory properties produced by enzymatic hydrolysis of crude egg white, which were mainly derived from ovalbumin. Among these peptides, two novel peptides with potent ACE-inhibitory activity were found, with amino acid sequences Arg-Ala-Asp-His-Pro-Phe-Leu and Tyr-Ala-Glu-Glu-Arg-Tyr-Pro-Ile-Leu.

Purified fractions from egg white protein hydrolysate showed several peptides identified as RVPSLM, TPSPR, DLQGK, AGLAPY, RVPSL, DHPFLF, HAEIN, QIGLF, HANENIF, VKELY, and TNGIIR, and investigated for angiotensin I-converting enzyme inhibitory activity, antioxidant properties, and anticoagulation activity [125]. In particular, the sequences ascribed to RVPSL, QIGLF, and TNGIIR exhibited high ACE inhibitory activity in vitro, with the IC50 value 20 lM, 75 lM, and 70 lM, respectively.

Hen’s egg white lysozyme-derived peptides showed moderate inhibitory activities against calmodulin-dependent phosphodiesterase (CaMPDE) and free-radical scavenging properties [126]. Egg lysozyme hydrolysates have potential as functional foods and nutraceuticals, although bioavailability studies are required to confirm their health benefits in humans.

## Figures and Tables

**Table 1:** Bioactive peptides in milk and dairy products.

Product	Carrier/Regulation	Peptide Sequence/Protein Fragment	Functionality	References
Milk	Endogenous enzymes	PYVRYL, LVYPFTGPIPN	ACE-I activity	[21,22]
	Protease from *Enterococcus faecalis*, enzymatic hydrolysis	LHLPLPL,αs1-CN f(90–94) (RYLGY), αs1-CN f(143–149) (AYFYPEL), and αS2-CN f(89–95) (YQKFPQY)	Antihypertensive activity	[22,23,25]
	endogenous enzymes	VLPVPQK	Antioxidant activity	[24]
	Proteinase of *Lactobacillus helveticus* PR4	Bovine αS1-casein; (αS1-CN) 24–47 fragment (f24–47), f(169–193), and β-CN f(58–76); ovine αS1-CN f(1–6) and αS2-CN f(182–185) and f(186–188); caprine β-CN f(58–65) and αS2-CN f(182–187); buffalo β-CN f(58–66);	ACE-I activity	[26]
		Ovine as2-CN fragments; f(165–170) LKKISQ, f(165–181) LKKISQYYYQKFAWPQYL, f(184–208) VDQHQKAMKPWTQPKTNAIPYVRYL, f(203–208) PYVRYL.	Antibacterial activity	[27]
		as1-casein f(1–23)	Immunomodulating activity	[28]
Cheese		Bovine β-CN f(13–28), αS2-CN f(5–21)	Mineral binding	[29]
*Manchego*		Ovine β-CN, fragment (199–204); α_s1_-CN f(102–109) KKYNVPQL; αs2-CN f(205–208) VRYL	ACE-I activity	[30,31]
*Emmental*		Fragments from as1_CN and β-CN	Immunostimulator, antimicrobial and ACE-I activity	[32]
*Gouda*		*α*_s1_-CN f (1–9), *α*_s1_-C f (1–13), *β*-CN f (60–68), *β*-CN f (109–111)	ACE-I activity	[33]
*Crescenza*		b-CN f(58–72)	ACE-I activity	[34]

**Table 2:** Schematic representation of processes generated for obtaining meat bioactive peptides.

Product	Process	Carrier/Regulation	Functionality	Peptide Sequence	References
Meat	Proteolysis, oxidation	Endogenous enzymes	ACE-I activity	APPPPAEVPEVHEEVH, PPPAEVPEVHEEVH, IPITAAKASRNIA, LPLGG, FAGGRGG, APPPPAEVP	[71,72,74]
	Enzymatic hydrolysis	Exogenous enzymes	ACE-I, antioxidant, antithrombotic, antimicrobial, and anticancer activity	KRQKYD, EKERERQ, KAPVA, PTPVT, RPR, GLSDGEWQ, GFHI, DFHING, FHG	[83,84,85,86,87,88,89,90,91]
	Cooking	High temperature	ACE-I activity	SPLPPPE, EGPQGPPGPVG, PGLIGARGPPGP	[72]
Collagen	Enzymatic hydrolysis	Bacterial collagenase, exogenous enzymes, protease from *Aspergillus oryzae*	ACE-I and antioxidant activity	AKGANGAPGIAGAPGFPGARGPSGPQGPSGPP, PAGNPGADGQPGAKGANGAP, GAXGLXGP, GPRGF, VGPV, QGAR, LQGM, LQGMH, LC	[92,93,94,95]
Cured products	Proteolysis	Endogenous enzymes	Antioxidant activity	DSGVT, IEAEGE, EELDNALN, VPSIDDQEELM, DAQEKLE, ALTA, SLTA, VT, SAGNPN, GLAGA, DLEE	[96,97]
Fermented products	Proteolysis	Presence of starter cultures	Antioxidant activity	FGG, DM	[98]
